# Drawing on Dialogues in Arts-Based Dynamic Interpersonal Therapy (ADIT) for Complex Depression: A Complex Intervention Development Study Using the Medical Research Council (UK) Phased Guidance

**DOI:** 10.3389/fpsyg.2021.588661

**Published:** 2021-02-18

**Authors:** Dominik Havsteen-Franklin, Mary Oley, Sarah Jane Sellors, Diane Eagles

**Affiliations:** ^1^Mental Health Services, CNWL NHS Foundation Trust, London, United Kingdom; ^2^Brunel University London, Uxbridge, United Kingdom

**Keywords:** depression, art psychotherapy, psychotherapy, complex intervention development, interpersonal

## Abstract

**Aim:** The aim of this paper is to present the development and evaluation of an art psychotherapy brief treatment method for complex depression for patients referred to mental health services.

**Background:** Art Psychotherapy literature describes a range of processes of relational change through the use of arts focused and relationship focused interventions. Complex depression has a prevalence of 3% of the population in the West and it is recorded that in 2016 only 28% of that population were receiving psychological treatment. This study was developed to test the hypothesis of whether an accessible and acceptable approach to the treatment of complex depression could be developed in relation to existing evidence-based practice within mental health services.

**Method:** The United Kingdom Medical Research Council phased guidance for complex intervention development was used (Phases I and II) to develop the intervention. The process included producing a literature overview, systematic description of clinical practice, including a logic model and a clinical protocol. The art psychotherapy protocol described an arts-based dynamic interpersonal therapy approach (ADIT), offered 1:1 over 24 sessions. Further to this the intervention was tested for referrer acceptability. The intervention is in the early stages of evaluation, using changes to the patient's depression and anxiety measured pre- and post-treatment with a follow-up measure at 3 months following completion of treatment.

**Results:** Phase I of the study provided a good basis for developing a logic model and protocol. The authors found that there was good clinical consensus about the use of a structured clinical art psychotherapy method (ADIT) and the literature overview was used to support specific examples of good practice. The verification of clinical coherence was represented by a logic model and clinical protocol for delivering the intervention. The acceptability study demonstrated very high levels of acceptability for referrers reporting that (i) ADIT was acceptable for patients with complex/major depression, (ii) that they were likely to refer to ADIT in the future (iii) that the use of arts was likely to improve accessibility (iv) the use of arts was likely to improve outcomes and (v) that offering ADIT was an effective use of mental health resources.

**Discussion:** Phase I of this intervention development study (following MRC guidance) demonstrated theoretical and practice coherence resulting in a clinical protocol and logic model. Whilst Phase II of this study showed promising results, Phase II would need to be sufficiently scaled up to a full trial to further test the intervention and protocol.

## Background

Approximately three percent of the Western population are diagnosed with complex depression (CD) and this figure continues to rise (World Health Organization, 1993). Socio-economic factors (de Mola et al., 2016; Agerbo et al., 2019), intimate relationships (Zlotnick et al., 2000), gender roles (Paykel et al., 2005), genetic and familial relationships (Sullivan et al., 2000) and the physical environment (Sarkar et al., 2018) are some of the factors that influence the development of CD. CD accounts for a significant socioeconomic burden, including the high prevalence of non-communicable diseases. For example, coronary heart disease, substance use disorders, hypertension, diabetes, kidney failure have all been identified as having a significant bidirectional influence (Chisholm et al., 2016). Further to this, the true impact of caregivers with CD on their child's development has not been fully accounted for nor has the full impact of non-adherence to physical treatment during episodes of CD. Chisholm et al. (2016) calculated a cost of depression to the global economy of $1.15 trillion and found that there was psychological treatment provided for only 28% of those patients diagnosed with CD in higher income countries. Complex depression is a broad categorization, given that the formal definition offered by the National Institute of Clinical Excellence (NICE, 2009) is based on a plethora of studies that are difficult to compare given their sample ranges and diagnoses (McPherson, 2020). For this reason, the authors identified CD according to key criteria that are shared across policy makers and can be observed in practice. The authors included the clinical screening criteria as feeling deeply sad, hopeless and out of touch with oneself and the world combined with other mental health issues or complex social issues that make the person particularly vulnerable (Caspi et al., 2014). This may be accompanied by feelings that life is not worth living and social isolation; episodic rather than generalized and persistent occurrence; lasting effects on mental and physical health and sequential comorbidity (Caspi et al., 2014). The ICD11 (ICD-11 - Mortality and Morbidity Statistics, 2020, p. 11) identifies the complexity of depression according to the severity of symptoms, including suicidality being present almost all day, every day for at least 2 weeks.

The psychobiological factors that link early trauma with neurobiological correlates has been well-researched (Yehuda et al., 1998; Sansone et al., 2001; Goodman et al., 2004; Cattane et al., 2017) indicating that the complex interplay between the biology and the psychology of the individual need to be taken into account to consider a full epidemiological appraisal. In recent years there has been an increasing interest in the preverbal, non-verbal and physical expression of relational experience to help articulate and mobilize communication and reflection within a social context (Schore, 2001). The identification of the relationship between physical sensations, actions, and their emotional counterpart, described as interoception, is also central to the data that is cognitively processed in CD states, resulting in misidentifications of feeling states and beliefs about self and other intentional states of mind [see Paulus and Stein (2010)]. Therefore, the authors posit that the use of art psychotherapy enables physical engagement and expression of emotional experience and that this is a core element of the dynamic process required to treat CD, especially where mental states and emotions may be difficult to grasp within the relational context. The authors propose that the use of art psychotherapy allows underlying unconscious body bound dynamics to be expressed, identified and reflected upon. Our hypothesis is that art psychotherapy results in a process that is both a bodily expression and a record of the process within a dynamic therapeutic context that can be readily accessed through engaging in the image-making activity rather than only through talking (Case and Dalley, 2014).

The authors rationale for developing this intervention was on the basis that diagnoses are commonly comorbid (Melartin et al., 2002; Sim et al., 2004) with complex etiology that require a range of evidence informed options to reach the most vulnerable (Kazdin, 2019).

## An Overview of The Literature

An overview of the literature examining arts psychotherapy as a treatment for depression was undertaken to consider and examine the efficacy of art psychotherapy approaches for people with CD. Predetermined themes for investigation were defined as: “arts-based,” “psychodynamic” and “effectiveness.” The authors used the following keywords: art psychotherap^*^ OR art therap^*^AND depression OR time limited OR dynamic OR interpersonal to identify papers in the following databases: EMBASE, The Cochrane Library, The British Association of art therapists (BAAT), University of East London Learning Resources (UEL), PsycInfo, and EBSCO (Psychology and Behavioral Sciences Collection).

### Arts-Based Art Therapy for Depression

The overview of the literature revealed that there were wide ranging theoretical frames of reference and practice principles. Most of these studies were brief group-based therapies, utilising group theoretical practices (Ponteri, 2001; Chandraiah et al., 2012; Zubala et al., 2017; Blomdahl et al., 2018; Ciasca et al., 2018; Ilali et al., 2018; Haslam et al., 2019). While a number of studies investigated dyadic approaches (Goldstein-Roca and Crisafulli, 1994; Trombetta, 2007), these were the exception. In the study by Blomdahl et al. (2016), half of the expert participants utilized psychodynamic theory in their approach. Humanistic, Developmental, Attachment, Object Relations, CBT and Integrative theoretical principles were also widely practiced, with Gestalt and TA models less widely so. A more explorative, phenomenological approach was taken in several of the studies (Trombetta, 2007; Chandraiah et al., 2012; Blomdahl et al., 2018; Ciasca et al., 2018). The shared theme for these studies was the art focused group work. Often the art focus was held in a supportive structure with an initial direction or theme provided, and space for verbal reflection, while the space for art making was openly explorative. For example, Zubala et al. (2017) took a structured approach and focused on person-centered interventions alongside psychodynamic principles. Ilali et al. (2018) also describe an art focus however is less openly explorative and more directive, with a specific thematic goal specified at the outset, while our study offered a synthesis of both talking therapies and creative approaches was trialed to create a new pluralistic “meta-approach” of therapy (Haslam et al., 2019). A predominant feature of the core practice principles within all of the arts-focused studies is the use of relational dynamics, whether within a group or dyadic relationship, as well as the use of art making to support expression.

### Psychodynamic Art Therapy Approaches to Treating Depression

The study by Zubala et al. (2013) ascertained that psychodynamic principles were the preferred theoretical approach for art therapists working with depression, and Barbee (1996) provides an example of, utilising psychodynamic principles with an arts focused approach in his study. The study by Thyme et al. (2007) was also based on psychodynamic principles within a time limited psychotherapy group, although this RCT provided comparative data with a verbal psychotherapy approach. While the survey provides insight into the broad range of practice principles that are adopted when working with this client group, Barbee (1996) and Thyme et al. (2007) apply relational principles to their approach, which correlates with the findings from the previous section, whereby focusing on relational dynamics was a consistent feature within the studies.

### Effective Art Psychotherapy Approaches

In 2013, Blomdahl et al., carried out a comparison of art psychotherapy approaches when working with people with depression. While there were no general conclusions to be determined due to the paucity of material, a suggestion that art psychotherapy can be undertaken successfully in a wide variety of settings, with both groups and individuals was indicated. In 2015 Korostiy and Hmain undertook a study on 150 patients with major depressive disorder (MDD). The study revealed a range of results dependent on the presenting symptoms, with an overall conclusion that art psychotherapy is an effective therapy in an integrated treatment of recurrent depressive disorder. A theoretical synthesis of group art psychotherapy literature (Gabel and Robb, 2017) as well as a systematic review considering the clinical effectiveness and cost-effectiveness of art psychotherapy among people with non-psychotic mental health disorders (Uttley et al., 2015a) included mood disorders such as depression and major depressive disorder in the eligibility criteria. Both demonstrated clear benefits of art psychotherapy as a treatment, as well as highlighting the need for further and ongoing critical analysis of group art psychotherapy practice and theory in the treatment of depression.

## The Aim of This Study

The aim of this study was to develop a cogent, evidence informed art psychotherapy brief intervention for CD guided by the Medical Research Council's (MRC) phased intervention development process (Shahsavari et al., 2020). Whilst there has been significant inquiry into the change processes (Zubala and Karkou, 2018) and manualisation of a psychodynamic art psychotherapy treatment model for depression (Blomdahl, 2017), as yet research within an NHS context that provides art psychotherapy treatment for CD that includes a clear logic model and pilot for this population has not been developed. This paper describes the work of an NHS based clinician researcher group (CRG) working with community out-patients with a diagnosis of complex depression where there was currently a gap in evidence-based approaches and where art psychotherapy was perceived to offer accessible and effective treatment. The predominant model of art psychotherapy for CD identified in the overview of the literature, has been driven by an understanding of relational dynamics, such as processing transference and countertransference phenomena and how these dynamics are intrinsic to dynamic representations produced in the artworks in art psychotherapy (Barbee, 1996; Zubala et al., 2014; Blomdahl, 2017; Zubala and Karkou, 2018). The secondary aim was to investigate where shared practice based principles were salient to a dynamic change process, including and in particular automatic and improvised expression (Keeney, 1991; Lobb, 2003; Blomdahl et al., 2013; Haslam et al., 2019), interpersonal articulation that allows naming and reflecting on complex emotional experiences (Gruber and Oepen, 2018; Zubala and Karkou, 2018) and developing narratives within a safe therapeutic relationship (Carlson, 1997; Bochner and Ellis, 2003; Gruber and Oepen, 2018). The second aim was informed by previous studies examining practice elements and change processes (see Havsteen-Franklin et al., 2016, 2017).

## Methods

When an area of mental health need has been identified that is not sufficiently met with existing resources, commonly an intervention is adapted through practice based evidence methods to achieve outcomes through applying an existing method to a similar population (Barkham and Mellor-Clark, 2003). The MRC has provided guidelines to assist intervention development through efficient utilization of collating and drawing on evidence. The CRG used these guidelines to examine the complex change processes in ADIT and the novelty of combining verbal structural elements and image making as core elements for the treatment of CD [see also Craig et al. (2008)]. The MRC intervention development protocol has 5 distinct phases (of which the first 2 phases were completed in this study):

Phase I: Theory development
Phase II: Modeling
Phase III: Exploratory Trial
Phase IV: Definitive RCT
Phase V: Long-term implementation

Theory development forms the foundation for modeling an intervention. The MRC allows a flexible theoretical development process within the phases, and the long-term aim is to develop effectiveness studies. The clinician-researcher group (CRG) for this study aimed to complete phases I and II of the MRC intervention development phases.

**Figure 1:**
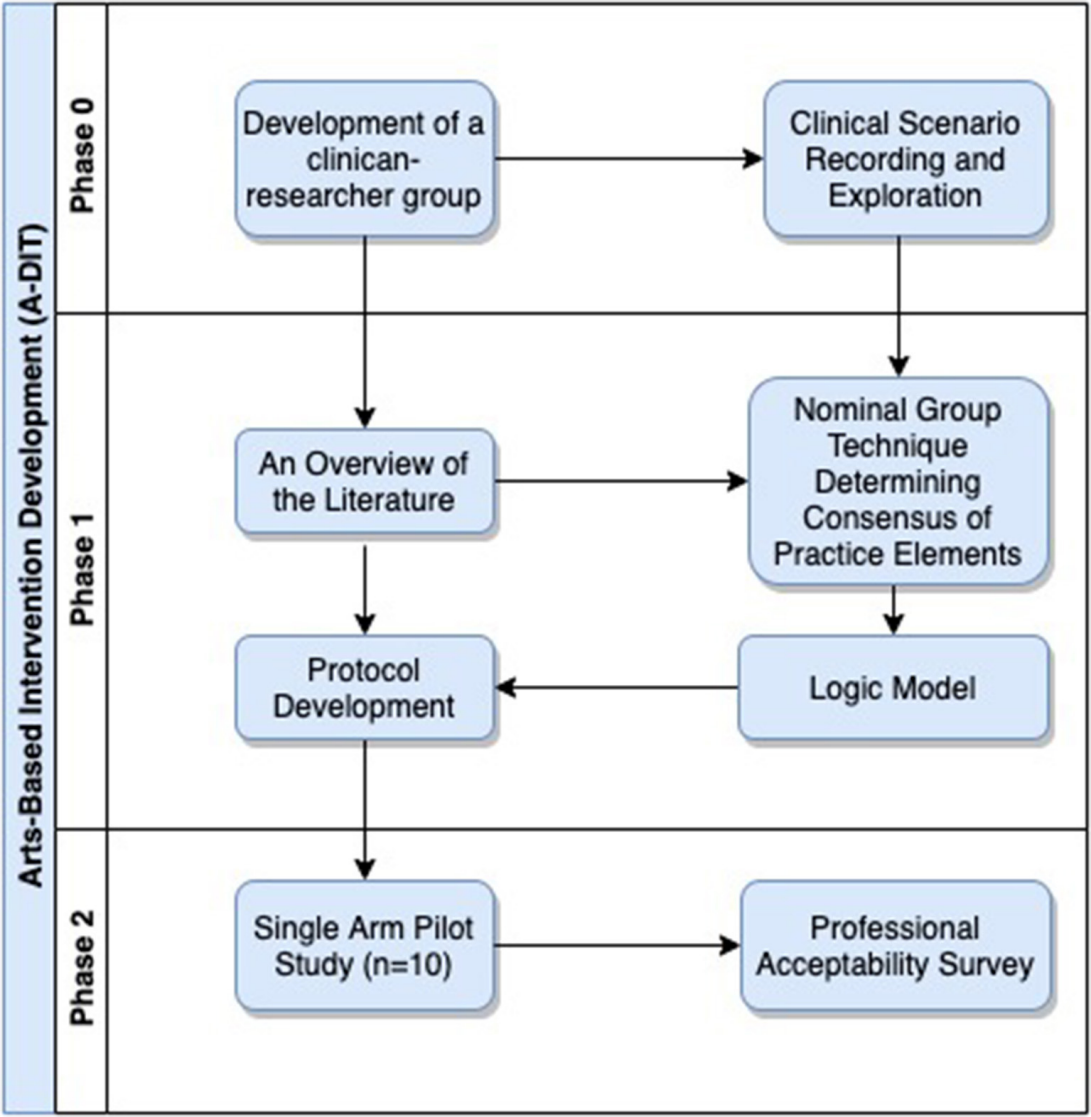
Structure of the intervention development process.

The CRG comprised experienced clinicians that were dual trained in Dynamic Interpersonal Therapy (DIT) and art psychotherapy. The group was co-chaired by a consultant in Arts Psychotherapies (first author). The CRG designed the study in three phases (Figure 1); phase 0 was peer exploration of CD clinical scenarios and exploration of whether there were shared principles of practice within art psychotherapy; Phase I was modeling the ADIT intervention through the use of a nominal group technique to support the theoretical development of a logic model and phase II involved designing a pragmatic single arm pilot with a professional acceptability survey of the ADIT intervention.

## Phase 0—Theory Development

The CRG Art Psychotherapists attended regular meetings to record role-plays, reflect on supervision and discuss the clinical interface between verbal and non-verbal actions in the art psychotherapy process using a DIT focus. The first author facilitated the sharing of practice principles and finding a common language to develop an integrated language and understanding of the ADIT process; providing a forum for clinical exploration that would lead to key terms for the overview of the literature, and protocol development. Three examples of ADIT engagement, clinical change and the use of the arts are described in short vignettes 1, 2, 3 below. These clinical examples describe the development of the model, integrating the structural elements of DIT within an art psychotherapy framework. The CRG were supported to think about the change process during group supervision with a DIT training lead from the Anna Freud Center. DIT and art psychotherapy are both informed by the theoretical understanding that a defensive processes can enable a level of functioning alongside relational disruption and complex depression (Lemma et al., 2011; Zubala and Karkou, 2018). The theoretical model follows that defenses are in place to prevent the reoccurrence of perceived or actual relational traumas or aspects of traumatic interpersonal events and that the interpersonal affective focus (IPAF) is embedded within an attachment-based system (Bouchard et al., 2008). The use of the image-making involved respecting, exploring, reconfiguring and developing a more flexible engagement with this defensive structure and supports to develop a therapeutic alliance. Further to this, the art form within art psychotherapy, enables a greater capacity for reflection and curiosity which are considered as central to the engagement process. In the initial phase of treatment, the art psychotherapist focuses on identifying repetitive relational patterns, the IPAF, which describes the interpersonal context of the presenting complex depressive symptoms. The second, phase of treatment aims to understand the person's role, affects and narrative within the relational pattern. Working through these relationship dynamics enables alternative ways of relating through reimagining self-other states within the therapeutic relationship. Finally, the last sessions focus almost entirely on ending and separation and reviewing the aims of treatment. Given the brief nature of the work, enabling exploration and emotional expression within the parameters of a dominant relational pattern linked with the depression, is considered important to the successful outcomes of ADIT. The hypothesis being tested by the CRG was the use of art making as a process to help exploration and expression, enabling an understanding of defenses, traumas and the relational pattern within the context of a brief intervention. The CRG explored a range of scenarios and concluded that the image-making process required sensitive facilitation as the image often encapsulated an aspect of the IPAF. Image making appeared to be an important addition to the DIT model where patients who were struggling to communicate their experience, were prone to disengaging, had problems with affect regulation and required tools to enable the imaginative appraisal of self and other mental states in narrative development. The findings from the case exploration and formulation were rich and the CRG felt that there were grounds to develop the model further and carry out a pilot programme in adult mental health services.

### Clinical Example 1. “Ms A”: Stage 1. Developing the Interpersonal Affective Focus (IPAF)

Ms. A was a 21 year old black British woman. She transitioned to adult mental health services in 2017 when she was 19 years old, having had previous support from CAMHS for depression, self-harm, suicidal thoughts and actions alongside low self-esteem and was considered to be vulnerable. Ms. A was a second child. Ms. A's mother was diagnosed with Schizophrenia and moved out of the family home when Ms. A was 9 and her sister 11. During her teens, Ms. A's sister had frequent admissions for Psychosis, A's father struggled to manage the children and was known to use drugs and alcohol, Ms. A was unsuccessfully placed with a number of foster families but frequently ran away, she eventually settled with a boyfriend's family although later spent time back with her father until finally being housed independently when she reached 18. After moving to adult services, Ms. A referred herself to Psychology, wanting help to “better understand” her moods, and “make sense of” her past, learn to manage the impact of her feelings. She considered that Art Psychotherapy might help and she took up the offer of the ADIT pilot.

**Figure 2:**
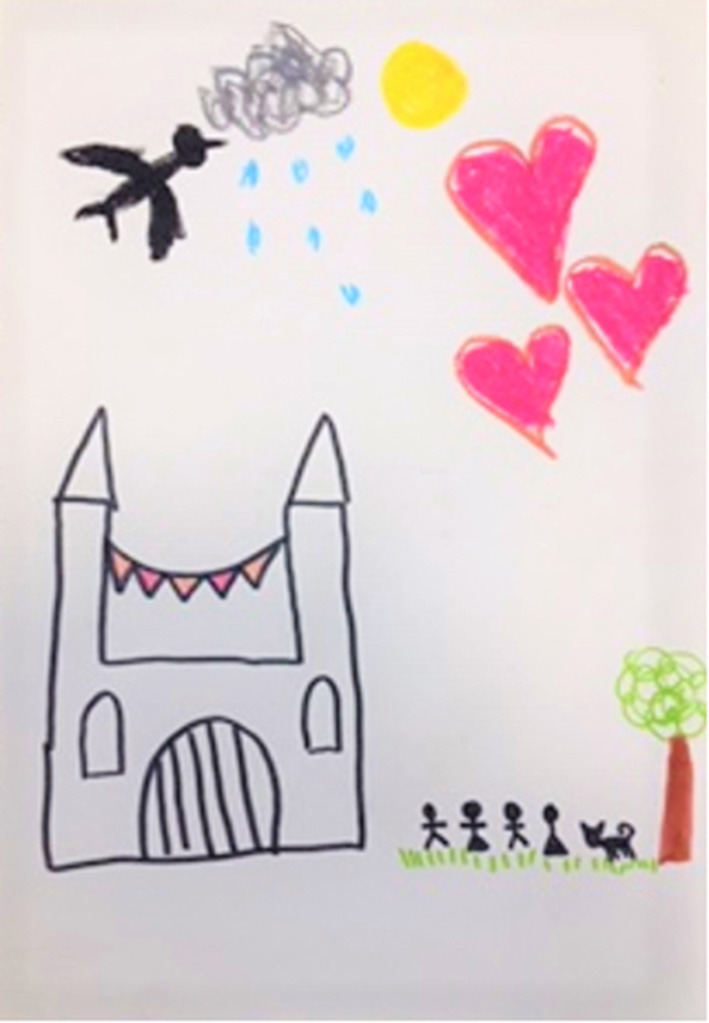
Image 1 (Ms A).

**Figure 3:**
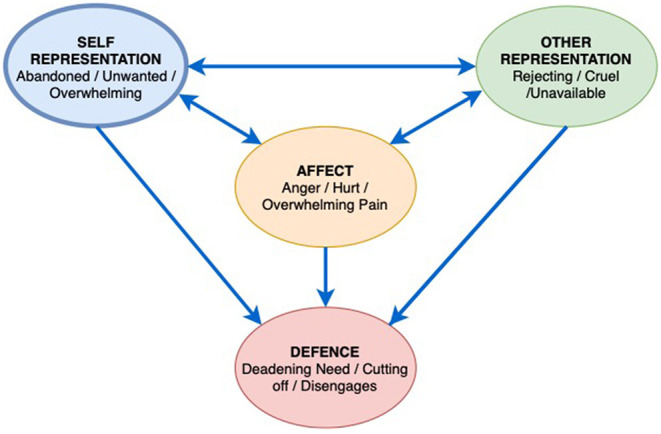
Patient Ms. A's Interpersonal Affective Focus (IPAF).

Figure 2 is from Ms. A's assessment for ADIT. It is a castle, with people and a dog outside with a bird, hearts, a cloud, rain and sun in the sky, which she alluded to as indicating a mix of emotions and moods. Ms. A explained that she is in the castle, with her family and boyfriend on their way to visit her. She has put up bunting between the towers to let them know that they are welcome. Ms. A's dog makes sure the family are travelling in the right direction toward her. Ms. A was aware of how she puts up huge protective barriers around herself and that this can make it hard for people who care about her, to know that she would like them to approach and that they are welcome. The image was an important early indicator of the Interpersonal Affect Focus other as rejecting, cruel and unavailable, self, feeling abandoned, unwanted, and overwhelmed, resulting in an angry affect with unbearable pain and hurt. Ms. A defends herself by cutting off emotionally and disengaging from others and help, deadening her needs (Figure 3) with this image the therapist was able to speak with Ms. A about the importance of defenses for her; her ability to be in control of these protective mechanisms; her desire to let people (including the therapist) know they are welcome, despite appearances, and the need for a split off part of herself, seen in the dog, to encourage this connection to occur.

### Clinical Example 2. “Mr. B”: On Not Making Art

Mr. B is a 60 year old man who took up a referral for the ADIT pilot following on from his engagement in art making during a hospital admission for a Manic illness with Psychotic features, he later presented with more chronic depression and anxiety. Although Mr. B felt his admission was linked to antibiotic medication, he also thought that there may be contributing historic interpersonal issues impacting on this. These came to light in the assessment. Mr. B believed that the art making might be a helpful way to express feelings which he found hard to speak about.

**Figure 4:**
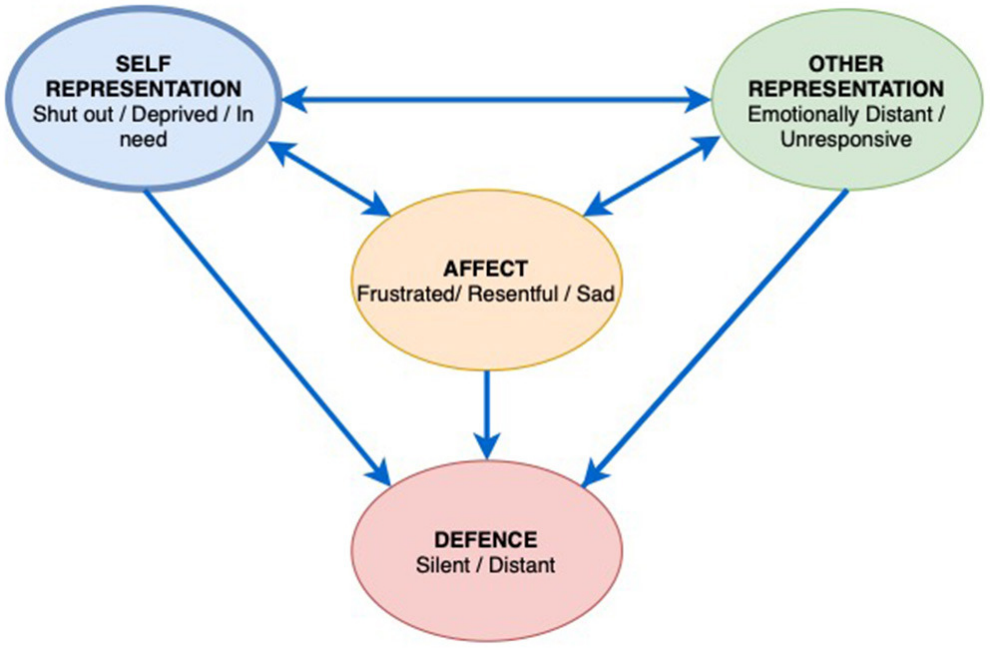
Patient Mr. B Interpersonal Affective Focus (IPAF).

Early on in the work Mr. B and the therapist identified a recurring pattern as the focus for the work, the way in which Mr. B felt shut out as a child, deprived and left in need. The interpersonal affect focus (IPAF) was linked to a cold and unresponsive father, and a mother who gave in a practical way but remained emotionally distant (Figure 4). Mr. B described a depressed childhood where his educational needs at school were not acknowledged. He now understands this was as a result of Dyslexia. Mr. B had a general feeling of being distant from others, their lack of warmth and misunderstanding resulted in him feeling resentful and frustrated at having to bear his emotional world on his own. In response, Mr. B would then deliberately distance himself, not ask for support and silence his need for connection and empathy.

**Figure 5:**
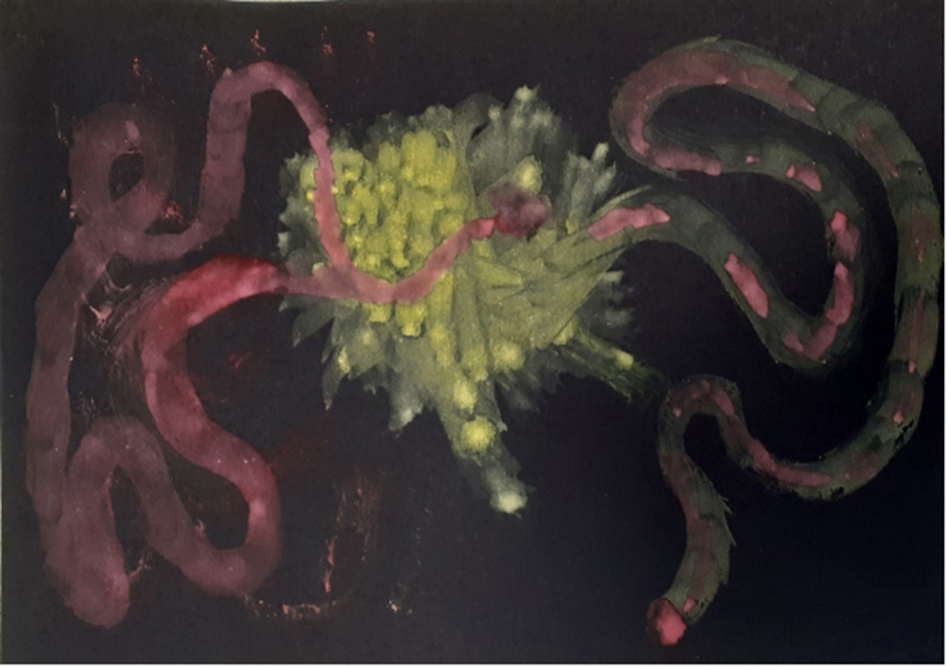
Image 1 (Mr B).

In the ADIT treatment Mr. B struggled to engage in art making. The therapist and Mr. B regularly explored the issues impacting on this; his depression affecting motivation; needing a direction from the therapist; the importance of him being able to verbally address his feelings. It was only in session nine when the therapist suggested again that art making might help him find a way to communicate his emotional experience, and allow a way to see his feelings, that he disclosed that he found the idea of the therapist watching him work unsettling. This situation echoed his IPAF where he had silenced his needs as he expected the other to be emotionally distant and unresponsive. To develop a collaborative approach in this context where the patient remained withdrawn and distant, the therapist attempted to alter the “relational pattern in a practical and concrete way, with the therapist asking would make a difference if I work alongside you?” Mr. B looked surprised and asked, “will you do that?” The therapist agreed, although this was not usual practice for her, but she recognized that making art together could be an important way of developing the therapeutic alliance and illustrating to Mr. B her understanding of his IPAF. Mr. B began to paint abstract forms on black that showed an uncertain journey and connection through a central shape, a simple but deceptively complex image of his journey to relate, and being hard to reach (Figure 5).

**Figure 6:**
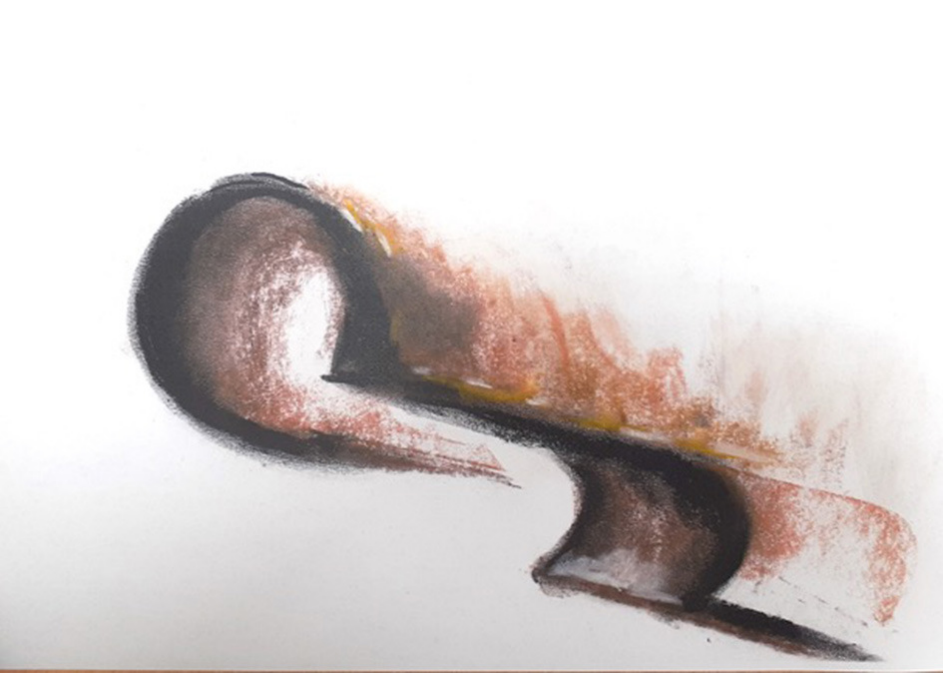
“Therapist's Image.”

The therapist's response was experienced as crucial to the therapy development. The therapist's image echoes the darker feelings expressed by Mr. B and creates a shape which highlights the internal and external, an uncertain pointed edged shape connects the two sides (Figure 6). Mr. B was moved by the therapists hearing and responding to his need to connect, this enabled the treatment to develop and move to a deeper level.

### Clinical Example 3. “Ms. C”: The Recurrence of Trauma Through Image-Making

**Figure 7:**
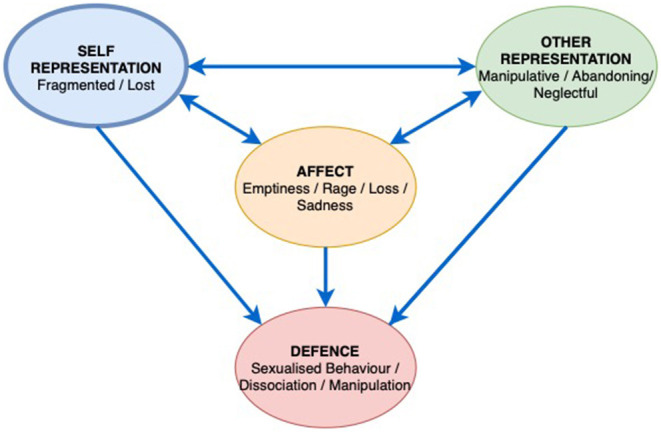
Ms. C's Interpersonal Affective Focus (IPAF).

Ms. C was a French Algerian, 43-year-old mother of one daughter, working in cosmetic retail at the time of her referral to ADIT. Ms. C's early years were populated with violent, unpredictable neglectful and abusive figures. Ms. C described often feeling overwhelmed with anger and frustration. Ms. C said that she was highly motivated to work on her difficulties, although she recognized that this might be challenging. When she described the people who had been close to her, she stated that her attachments to others was experienced as fragmented and untrustworthy. When she attempted to defend against the pain of those chaotic attachments, she was left with a sense of abandonment, emptiness, sadness and loss prompting her to seek care giving figures and commence the cycle again. One way that Ms. C managed her anxieties was through sexualising relationships. The therapist became aware that this unconsciously repeated the abusive attachment patterns of her childhood. This sexualised behavior was often was accompanied by an experience of dissociation. Ms. C felt that she was good at adapting to others needs, trying to fit to whatever the other person wanted of her; although she would then often feel she had lost herself, and that no one could understand her. When overwhelmed, Ms. C would cut off from her emotions and feel like she was in a “bubble,” a protective place but one which prevented her from feeling. This ultimately led to severe depression and dissociation. These dynamics constituted the underlying principles of her interpersonal affective focus (Figure 7).

**Figure 8:**
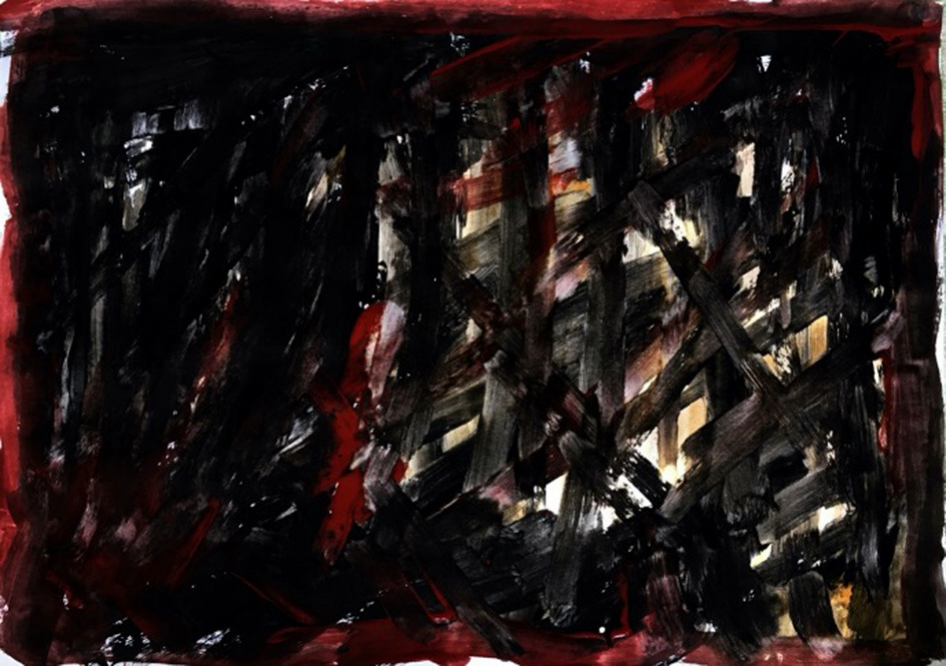
Image 1 (Ms C).

**Figure 9:**
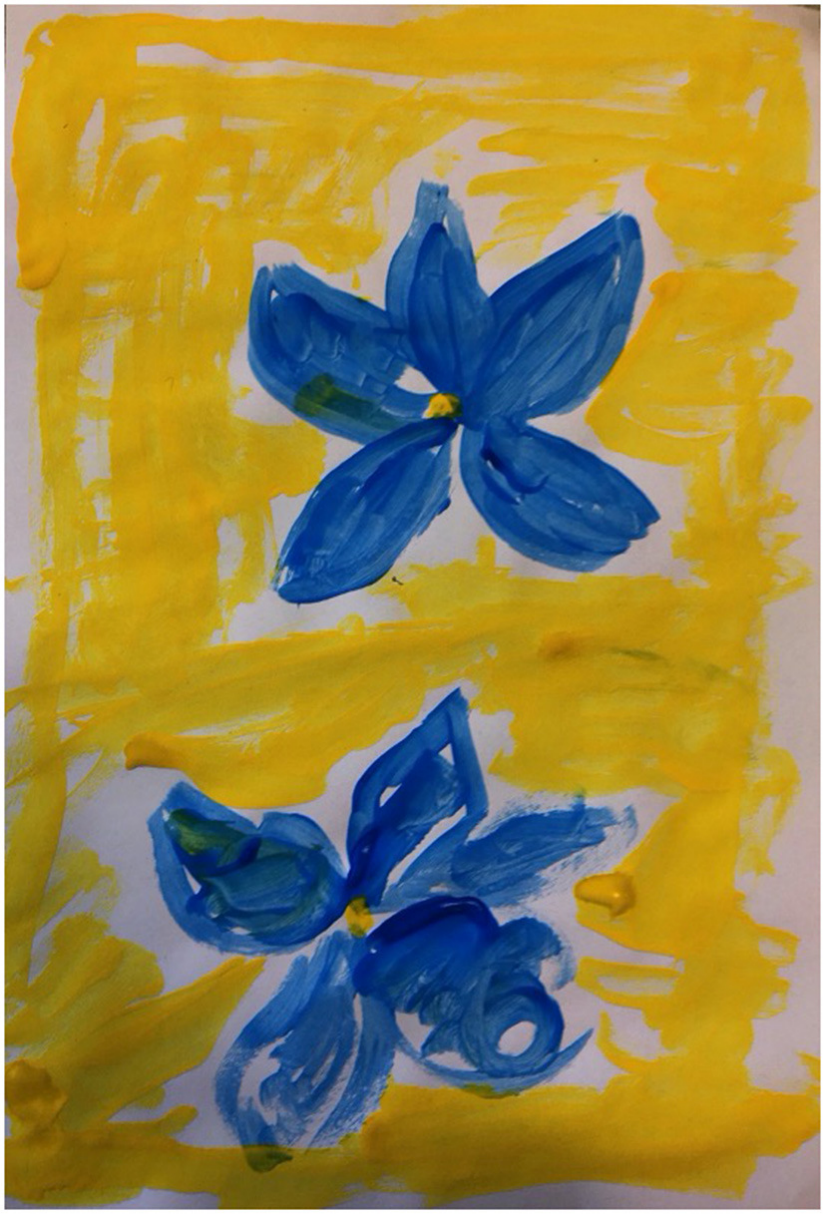
Image 2 (Ms C).

Ms. C was motivated to make and use imagery; retrospectively describing her paintings as “a violence,” (Figure 8) which confronted her with fragments of the past. She produced several images of dresses which she recalled wearing during traumatic and significant times in her life. These were painted as abstract fragments of patterns (Figure 9). The fragmented stories and images powerfully communicated her feelings of fragmentation, dislocation and confusion through the countertransference. The image of a treasured yellow dress with blue flowers evoked memories of a summer day when driving with friends. Ms. C, in the passenger seat, described the driver falling asleep and the car plunging into a ravine. She remembered the dress being covered in blood and the doctor commenting regretfully that she would have to cut the dress to treat her, powerfully evoking the trauma experience in her need for help.

**Figure 10:**
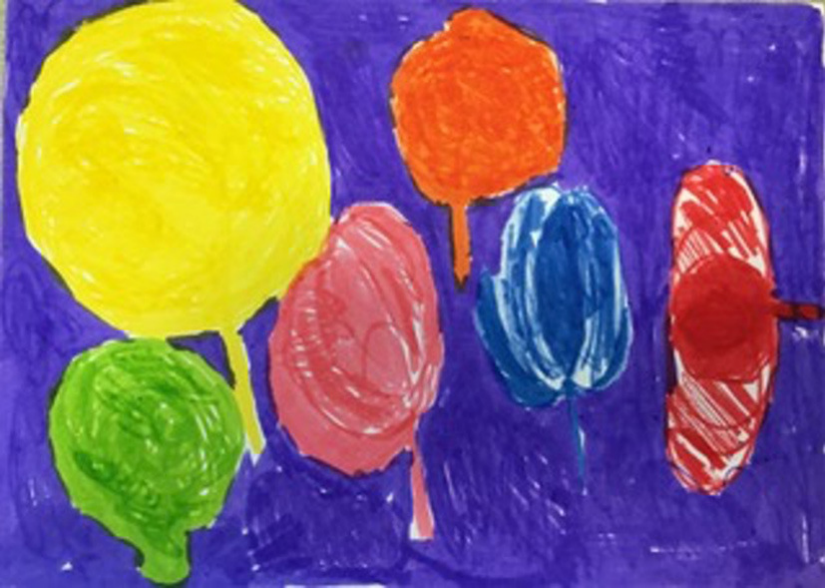
Image 3 (Ms C).

In a later image, Ms. C represented internal body parts with several balloon shapes of different sizes (Figure 10). The lack of connection between the organs, which looked as though they could float off at any moment, resonated with deeper structural and relational deficits. Ironically, this was the most coherent representation Ms. C produced of her dissociation. The countertransference during treatment was an important part of the work in bearing her projective sense of fragmentation and confusion connected to early chaotic object relations. The art making offered a vital space in which to safely project fragments so that they could be referred to and held safely between Ms. C and the therapist. At the ending of the work, Ms. C was more able to identify a range of feelings. Concomitantly her depression intensified although she noted a decrease in her anxiety, panic attacks and nightmares. Ms. C said she no longer wished to work in cosmetics, instead she wanted employment in the caring professions. The therapist and Ms. C considered how the involvement in the world of cosmetics served as a protection from the “ugly” and uncomfortable “reality” which she had been committed to confronting and understanding during her therapy and the development of a manageable relationship to reality.

## Phase 1—Intervention Modeling

The clinicians that were invited to be part of the modeling phase were chosen using purposive sampling based on the following inclusion criteria; being an experienced art psychotherapist (more that 10 years of practice); having a good understanding of psychodynamic processes in art psychotherapy and either a training in Dynamic Interpersonal Therapy with the Anna Freud Centre, London, or in the process of completing it.

Using peer supervisory sessions and explorative roleplay, the CRG shared consensus on approaches to working with the dynamic change process. These approaches were primarily based on the use of arts to enable the patient's intrapsychic systems for managing painful events and experiences, that resulted in interpersonal defenses to be safely explored within an interpersonal context. Having identified a psychodynamic change process, the lead investigator invited participants to engage in a nominal group technique (NGT) exercise (*n* = 5).

The NGT has also been used in healthcare contexts to develop consensus through a staged group process identifying key concepts where there is ambiguity (Havsteen-Franklin, 2014) for example about professional competence (Steward, 2001) meeting patient needs (Peña et al., 2012) practice based evidence (Rankin et al., 2016) and exploratory health studies (Van de Ven and Delbecq, 1972). The NGT has been used as a method for identifying best practice where there are gaps in evidence-based treatment (Rankin et al., 2016). The method uses ranking within a face-to-face focus group type setting. Unlike many focus group methods, which usually facilitate an exploration of a question throughout, the NGT is a methodology that employs a structured four-stage process that provides a reliable transparent strategy for eliciting participant responses and enabling participants to make prioritisations both as an individual and a group. Having already covered significant ground during open group explorations, the NGT was used primarily as a method to consolidate complex ideas about a dynamic change process and what core competencies should be prioritized in a health context. Whilst other methods, may be used for clinical modeling, for example the Delphi methodology, which uses a reiterative process of theme identification, the NGT is a more time efficient tool. The moderator of the group begins by identifying the question being asked and whether all participants understand the question. Following this the moderator asks the participants to write their responses in brief statements and ideas. The moderator directed participants to respond to the question “What are the constructs used in an arts-based dynamic interpersonal model that are required to impact on complex depression within a mental health secondary care context?” Each person wrote down their response on paper provided and then the moderator requested the participants to share them in a round robin style of feedback. The next part of the session included clarification, summation and deletion of replication or redundant constructs. Constructs were defined as elements integral to the dynamic process and were facilitated by the art psychotherapist. The group continually refined the constructs to produce statements that satisfied all participants. Where there was a significant difference of opinion the construct was discussed and reworked until all members felt the wording was reliable and valid according to the clinical process. The NGT resulted in the following change process constructs:

1. Activating emotional expression
2. Containing affect
3. Stimulating/modeling mentalizing/play/free association/imagination
4. Re-visioning the interpersonal affective focus (IPAF)/therapeutic narrative
5. Physically engaging in expression
6. Versatile/flexible approach to enabling a range of expressions from illustrating to embodiment either implicitly or explicitly
7. Managing relational proximity
8. Allowing unconscious to be explicitly and slowly elaborated upon whilst defenses are implicitly maintained (as needed).
9. Safely moving between concrete and symbolic expression
10. Highlighting “here and now” relational processes
11. Enabling motivation and decision making to develop a sense of agency
12. Shared experience/witnessing as metabolizing and developing a shared language/epistemic trust
13. Facilitates use of free association/imagination.

**Figure 11:**
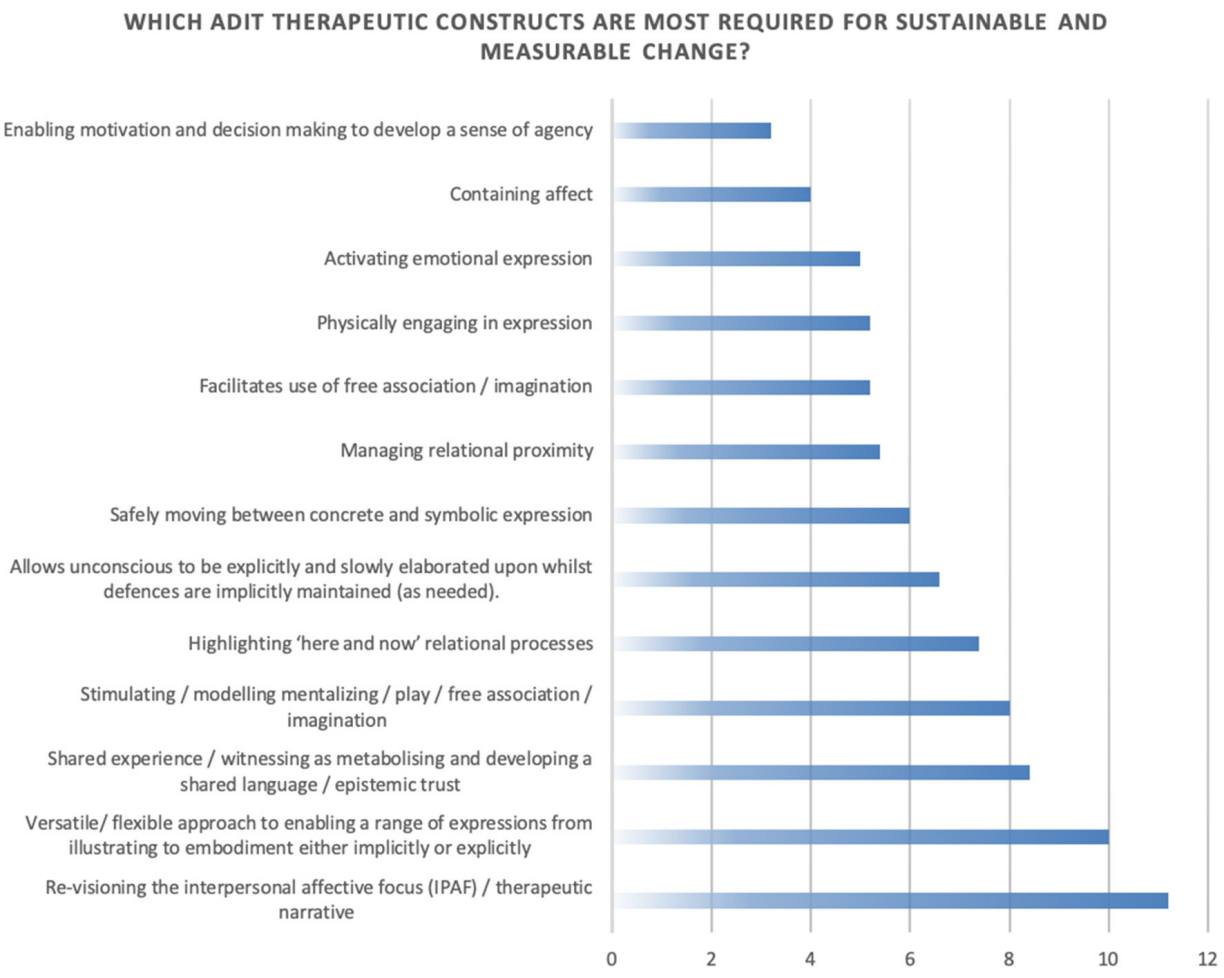
NGT consensus on constructs underpinning ADIT.

In the last stage of the NGT, in order to determine a stronger potential relationship between complex depression and the constructs, the moderator asked the participants to rank which constructs they felt were “more required” for achieving measurable change with complex depression (see Figure 11). This process took into account the use of outcome measures, duration of treatment, competencies required, and the sustained reduction of symptoms.

The ranked results of the NGT group process described two areas of change that were considered being more required for an impact upon complex depression symptoms; firstly, identifying and re-visioning conscious and unconscious relational dynamics and affect and how these related to specific interpersonal patterns; secondly that a versatile use of arts was required to mobilize this change. The findings indicated that the group considered several approaches within the framework to be of primary importance to working with depression; relational reworking through exploratory image making as a way to imagine other ways of relating within interpersonal narratives and the use of arts to illustrate, embody and express their experience. Constructs that were considered less relevant were *containing affect* and *enabling motivation and decision making to develop a sense of agency*. These findings were congruent with the descriptions of the clinical process (see Clinical Examples 1–3). It was evident that the findings from this group illustrated a close valence to existing studies showing a strong emphasis on exploring relational dynamics (Barbee, 1996; Zubala et al., 2014; Blomdahl, 2017) and there being an expressive arts focus to enable exploration of the relational content (Chandraiah et al., 2012; Blomdahl, 2017; Ciasca et al., 2018).

DIT was used as a comparative intervention template for the new intervention. Due to the focus on depression, training and clinical experience of the NGT participants, it is not surprising that most of the clinical elements, such as referral criteria, aims of treatment, outcome measures, referral and exclusion criteria, assessment and phases of treatment were considered to be very similar. However, it was noted that the use of art materials was a novel addition to a DIT model which influenced how the process unfolded. Further to this, there was more emphasis on the narrative development as was verified in the NGT group. Once the CRG had an opportunity to reflect on each of these areas, the findings were assimilated within a clinical protocol. Table 1 describes the sections of the protocol and the main similarities and differences between a DIT and Art Psychotherapy approach to the treatment of depression.

**Table 1:** Protocol elements.

**Protocol elements**	**Description**	Protocol element present in Dynamic Interpersonal Therapy Model (DIT)^*^
Diagnosis	Severe Depression symptoms as described by the ICD10 combined with social vulnerability.	No
Aim of treatment	The aim of the treatment is to provide a better understanding of how the patient's relationships function, their interpersonal narratives, and for the patient to test alternative ways of relating.	Yes
Context of treatment	Secondary Care Mental Health Services	No
Outcome measures	PHQ9/GAD7	Yes
Duration of treatment	24 Weeks	No
Referral criteria	• History from GP or referrer including physical health. • Risk Assessment from referrer. • No PTSD symptoms present. • No current excessive substance misuse (impulse control). • No primary diagnosis of BPD. • No active psychosis. • Motivated to use the arts in a therapeutic context. • There should be evidence that the depression is caused, maintained or exacerbated by interpersonal relating. • There should be evidence of motivation and a capacity to tolerate ambiguity and frustration. • There should be sufficient ego-functioning to sustain and make use of the process in the therapy and in the wider context. • To have a caring, consistent and reliable relationship.	Yes
Exclusion Criteria	• Florid psychosis. • Actively suicidal with intent. • No social contact (e.g. family friends colleagues). • Homelessness. • No motivation to receive therapy. • Substance/ alcohol misuse as primary diagnosis.	Yes
Assessment	• Dynamic formulation. • Risk assessment and crisis plan. • Exploring the relationship between symptoms and interpersonal context. • Exploring the use of arts. • Exploring use of the session, engagement, and readiness for treatment.	Yes (not including the use of art)
Phase 1 of ADIT	Approximately 6 weeks focusing on identifying and mapping the interpersonal affective focus (IPAF) within the narrative contexts, including triggers and related relational patterns and understanding the symptoms of depression in relation to the IPAF.	Yes
Phase 2 of ADIT	Approximately 12 weeks following Phase 1 to explore the underlying affect, defenses, role, triggers and identifying new ways of relating within the IPAF in the intra and interpersonal contexts.	Yes
Phase 3 of ADIT	Approximately 6 weeks following Phase 2, ending at 24 sessions. Understanding the impact of the ending, fantasies about the ending and how these manifests within interpersonal contexts. The authors introduce an ending letter within Phase 3.	Yes
Follow Up	The art psychotherapists offered up to three supportive follow-up sessions within 6 months of ending.	No

* *Note that since the development of the ADIT protocol, a verbal model of DIT has been adapted for complex care (DITCC) (Rao et al., 2019)*.

**Figure 12:**
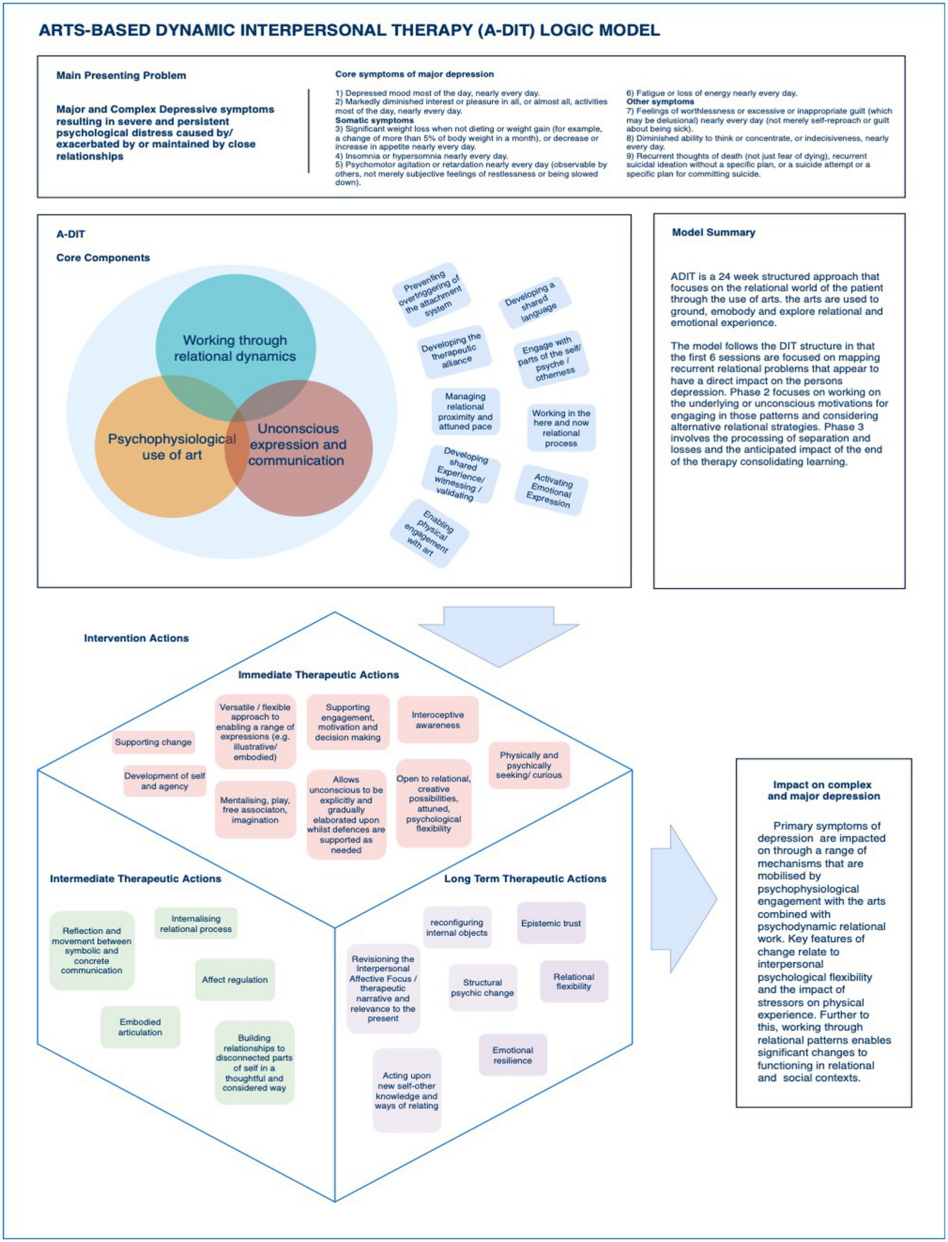
ADIT logic diagram.

Given that the key adaptation of DIT for practice by experienced art psychotherapists was the inclusion of image making as the core clinical process, the CRG discussed the change process with the addition of image-making processes and decided on the resulting acronym offering an accurate representation of the model; Arts Based Dynamic Interpersonal Therapy (ADIT) The chair of the CRG drafted a description of the clinical process that reflected outcomes of the NGT, carried out the overview of the literature, and chaired the exploratory discussions. Three iterations of a logic model were made that responded to changes from CRG members, especially the “Core components” of the model being accurately worded and the intervention actions which accompanied these (see Figure 12).

## Logic Model

A logic model is a versatile visual tool, used to describe an overview of the major practice elements where there is consensus on the perceived problem, process and outcome of an intervention or programme (Julian et al., 1995). Our logic model (Figure 12) begins with the presenting issues related to CD. Following this, the core intervention components are described and the subsequent actions that result from the facilitation of those elements. The core components were *working through relational dynamics, psychophysiological use of arts* and *unconscious expression and communication*. The box form (bottom left of Figure 12) shows the immediate, intermediate and long-term impact of the model and the interrelatedness of the perceived impact. The final outcome relates to the impact on the original problem.

## Phase 2—Pilot Study

Phase 1 described the theoretical modeling and the process of the proposed intervention as an assimilation of two models of treatment, DIT and art psychotherapy resulting in a blended model of Art-based Dynamic Interpersonal Therapy (ADIT). The pilot study was designed firstly to investigate the fidelity of treatment described in the protocol and secondly to investigate how the intervention impacted on CD with a small cohort.

### Recruitment

Patients were recruited from secondary care NHS community teams in an urban environment, where the patient met the criteria for CD (see Table 2). As a brief treatment for patients with a primary diagnosis of CD was new to the services, the uptake was over a period of 1 year. The demographics showed that the majority of participants were women (7/10) single (7/10), with a mean age of 35 years and a range of 21–60 years old. Only one patient held a degree and 5/10 held other qualifications.

**Table 2:** Sociodemographic characteristics of the patients included in the pilot.

**Sociodemographics characteristics**	**(*N* =)**
**Sex**	
Male	3
Female	7
**Age**	
Mean (sd) Range	35 (14.9) 21–60
**Ethnicity**	
White	6
Black African	4
**Marital status**	
Married/Co-habiting	1
Single/unmarried	8
Not stated	1
**Qualifications**	
Degree or equivalent	1
**Higher education qualification**	
A-Level or Equivalent	1
GCSE	3
Technical qualification	1
No formal qualifications	2
Not stated	3
**Employment**	
Full time	2
Part time	2
Unemployed	5
Not stated	1
**Currently taking anti-depressants**	
Yes	5
No	5
**Baseline Measurements**	
PHQ9 Mean (SD) Range	20.7 (3) 18–27
GAD7 (SD) Range	15.6 (5) 7–26

**Figure 13:**
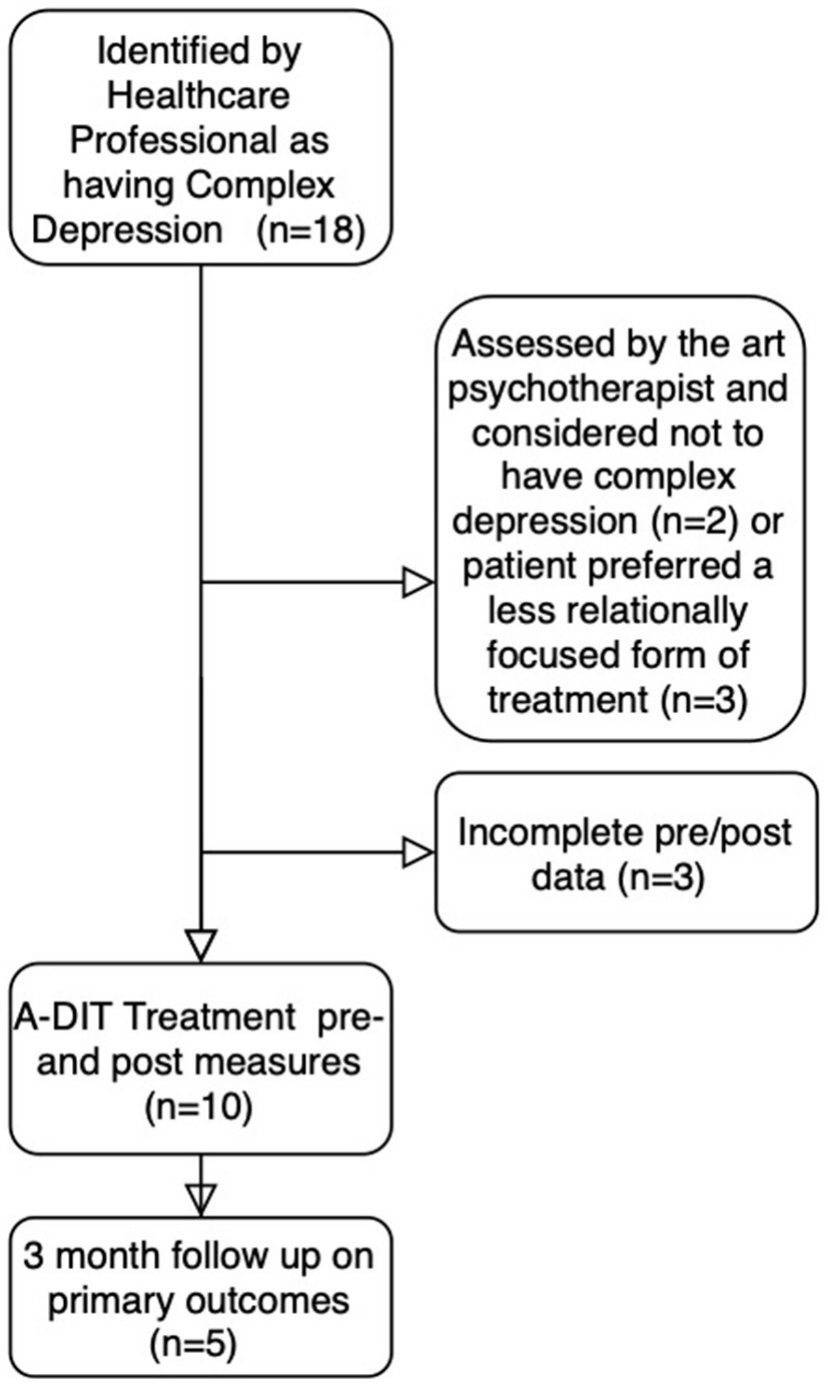
Consort diagram.

The pilot was authorized and ethical approval given by CNWL NHS Foundation Trust and Patients were recruited and assessed by the CRG members. The pilot was registered with CNWL as a Quality Improvement project describing changes to service provision. Weekly meetings were arranged to discuss referrals, assessment and treatment methods as well as adherence to the protocol. Early in the process the authors addressed the way that the assessment was being conducted as some patients were anxious about the process exposing them to interpersonal difficulties in ways that activated unmanageable patterns of defense and vulnerability. The CRG explored the assessment criteria and made adjustments to the protocol, accordingly, adjusting the information sheet using plain English language and allowing more sessions for the assessment process. The total number of patients referred was 18, however 3 patients dropped out, 2 were considered not to have complex depression at the point of assessment. A further 3 patients chose not to complete the outcome measures (Figure 13).

To monitor treatment fidelity, weekly meetings were held by the CRG to explore practice in relation to the protocol. Clinicians agreed that ADIT was delivered according to the protocol summarized in Table 1. with some minor variations that were considered to be congruent with the specifics of the complex presentation, for example, as described in the first case example, the initial work was sometimes more verbal as the patient struggled to engage in image making. This contrasted with the third case example where the patient readily used the image making to explore affect, associations and memories. Further to this, in some scenarios, there was greater elaboration on the sequences of interpersonal events that constituted the interpersonal affective focus whereas at times it was more focused as illustrated in case examples 1 and 2. As well as weekly meetings, monthly clinical supervision and protocol adherence, support was provided by an experienced DIT psychoanalytic psychotherapist who also developed DIT for complex care (Rao et al., 2019) and trains DIT practitioners at the Anna Freud Center, London. The CRG also continued to have their own individual art psychotherapy supervision on a monthly basis.

The primary outcome measure for symptoms of depression was the Patient Health Questionnaire−9 (PHQ-9). The scale is a validated and reliable measure that is widely used to measure symptoms of depression in healthcare contexts (Löwe et al., 2004). The optimal cut-off score is 10 in most conditions (Manea et al., 2012). A second brief measure for assessing Generalized Anxiety Disorder (GAD-7), was used as symptoms of anxiety commonly accompanies severe depression in at least 50% of the population (Fava et al., 2000) and appears to be correlated with psychosocial functioning (Rodriguez et al., 2005). GAD-7 is a widely used measurement instrument for anxiety in outpatient contexts (Rutter and Brown, 2017, p. 7).

### Effectiveness

**Figure 14:**
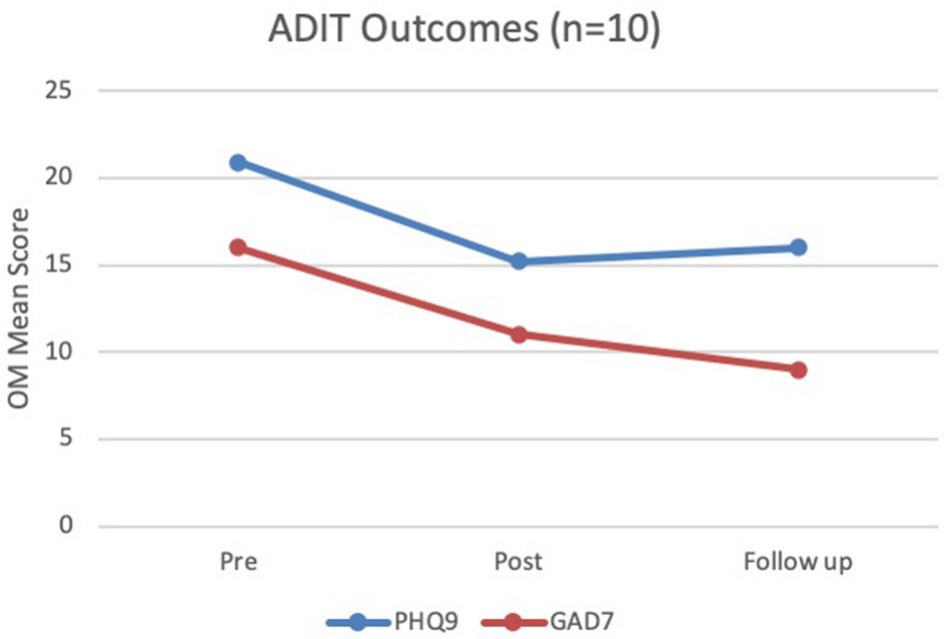
Pilot outcomes (*n* = 10).

In this study the sample size was small (*n* = 10) and therefore the statistical power was low, and the confidence intervals were wide. All patients were requested to complete pre- and post-treatment measures and were also seen 3 months after treatment for a follow-up session. The sample is small, however the results are consistent with trends in verbal psychological therapies treatment for depression (Saunders et al., 2019). The results (Figure 14) indicated good outcomes also consistent with the recent outcome studies being developed for verbal interventions for CD (Rao et al., 2019). For the Patient Health Questionnaire (PHQ-9), scores of 5, 10, 15, and 20 represent cut-off points for mild, moderate, moderately severe and severe depression, respectively. Sensitivity to change has also been confirmed. The total score ranges from 0 to 24. The mean pre-treatment score for CD indicated severe depression, reducing to moderately severe after 24 weeks with a slight increase still in the same range of moderately, but not progressing to severe, suggesting an overall improvement with only slight increase at 3 months post-treatment. The measure used for identifying changes to generalized anxiety disorder was (GAD-7). GAD-7 is scored similarly where 5, 10, and 15 represent cut-off points for mild, moderate and severe anxiety, respectively. Interestingly, ADIT didn't focus on anxiety specifically, but there appeared to be better outcomes in this domain. The average scores for the GAD-7 scale pre-treatment were measured as severe and reduced to moderate by end of treatment (24 weeks) and there appeared to be symptom reduction maintained at 3 months post-treatment where the average score indicated mild symptoms of anxiety.

### Mental Health Professionals Survey Responses on the Acceptability of ADIT

The role of the mental health professionals and their understanding of effective interventions is important to the development and scalability of any health intervention. According to recent research, referrer confidence in an intervention being acceptable correlates with patient take-up (Cruz et al., 2008; Levy and O'Hara, 2010; Mohr et al., 2010; Titzler et al., 2018). During the pilot phase of the project, the authors administered a survey to investigate referrers acceptability of ADIT, based on their experience of the referral process and knowledge of ADIT. The authors also asked whether ADIT was acceptable in terms of perceived benefit to patients and whether ADIT was perceived to be a good use of NHS resources. The project received Quality Improvement study status within the NHS and was ethically approved by local NHS ethics. The authors also asked questions about the parameters of the pilot being for patients with complex depression in secondary mental health community services and whether they agreed with the limitations. The total number of respondents was *n* = 37 and the results demonstrated a high level of acceptability of this intervention within a mental health service.

A sample of 61 subjects across mental health services in central and north west London were identified having referred patients with CD within the past year. This included arts therapies services. Each subject was notified by email and asked to complete a Google Form by clicking on a link in the email. Information about the data use and consent was provided at the beginning of the survey. The survey included demographic information and the questions were designed using an adapted version of Kazdin's Treatment Evaluation Inventory (Kelley et al., 1989) to include the following questions using a Likert type bipolar scale where 1 = *I strongly do not agree* and 5 = *I strongly agree*.

1. ADIT is an acceptable form of treatment for patients
2. I am likely to refer patients to ADIT in the future
3. ADIT is likely to be acceptable to patients with complex/major depression
4. The use of arts are likely to improve accessibility
5. The use of arts are likely to improve outcomes
6. Offering ADIT is an effective use of mental health resources
7. ADIT should only be used with patients with complex depression
8. I would recommend this treatment to a friend or family member with complex depression
9. ADIT should only be piloted within secondary care community services.

Thirty seven surveys were returned which is a 60.66% response rate. The respondents were psychological therapists (including arts therapists) (*n* = 34) and psychiatrists (*n* = 2) and a nurse (*n* = 1) based in community adult mental health services. In the survey, conducted between March–July 2020, 29 respondents (78.3%) agreed or strongly agreed that ADIT is an acceptable form of treatment and 25 respondents (67.56%) agreed or strongly agreed that ADIT was acceptable for patients with complex/major depression, and 25 respondents (67.56%) agreed or strongly agreed that they were likely to refer to ADIT in the future. Further to this 30 respondents (81.08%) of referrers stated that they agreed or strongly agreed that the use of arts were likely to improve accessibility and 26 respondents (70.26%) agreed or strongly agreed that the use of arts were likely to improve outcomes. Thirty respondents (81.08%) agreed or strongly agreed that offering ADIT was an effective use of mental health resources. Similarly, the majority of respondents also answered the friends and family test positively where 21 respondents (56.75%) agreed that they would be likely to refer a friend or family to ADIT and 16 respondents (43.25%) were not sure. The specificity of the intervention being provided only within secondary care community services had mixed results with 9 respondents (24.32%) agreeing or strongly agreeing, 16 respondents (43.25%) being not sure, and 12 respondents (32.43%) disagreeing or strongly disagreeing that ADIT should only be piloted within secondary care. Similarly, there were a range of responses for whether ADIT should only be used for patients with complex depression with 9 respondents (24.32%) agreeing or strongly agreeing, 19 respondents (51.36%) being not sure, and 9 respondents (24.32%) disagreeing or strongly disagreeing that ADIT should only be used for complex depression. The main findings from the survey were that from mental health referrer perspectives, ADIT was a highly acceptable intervention and that there was a high degree of confidence in art improving accessibility and outcomes for patients with a diagnosis of CD.

## Discussion

The advancement of an accessible and acceptable evidence-based intervention for complex depression would be a significant step for vulnerable populations who are not accessing psychological help. This study described a phased approach to intervention development following the MRC guidelines including empirical data regarding the modeling and effectiveness of a brief arts-based dynamic interpersonal intervention. A key component of the modeling of the intervention was drawing upon evidence already established within systematic reviews and pilots and using this to support the development of ADIT. Whilst many of the practice elements appear to be transdiagnostic [see Havsteen-Franklin et al. (2017)], dosage is less researched as is the optimum format of delivery. The overview of the literature described Art Psychotherapy as usually offered for complex depression in a group format for a variable period of time. This study researched a novel approach of providing 24 weeks of individual ADIT in secondary care and using a structure that is closely related to an evidence based verbal intervention (DIT).

The strength of this study was the development of an intervention through a collective consultative approach within a working clinician researcher group committed to defining practice and building on existing evidence. Within the first phase of this study (Phase 0), prior to the logic model development, the authors explored a cogent theoretical model with the evidence informed rationale that key relational factors influence the etiology, maintenance or exacerbation of complex depressive symptoms (Beck and Alford, 2009; Lemma et al., 2011). To this effect the qualities of attachment and separation, interpersonal dynamics and unconscious affects were primarily explored through the image-making process. For example, often memories and affects became vivid in the image-making process, helping to contextualize anxieties and trauma reactions. Further to this, the interoceptive emergence of affects through the arts form helped to shed light on feeling states and intrapsychic relational dynamics contributing to insecure attachment styles. It was also apparent that image making needed careful relational facilitation, respecting and attuning to defenses as not to overstimulate the attachment system or evoke unmanageable trauma reactions. Additionally, in some circumstances during ADIT treatment, talking became the principal method of facilitating the therapy, especially where image making was felt to be too threatening or exposing. This meant that the CRG required the art psychotherapist to have a good knowledge of how psychodynamic knowledge and practice competence can facilitate dynamic interpersonal therapy through verbal and arts-based means.

The authors advise cautious interpretation of the findings due to the lack of comparability with other interventions, the small scale represented by the pilot data and unknowns about the scalability of the intervention beyond the representative group of clinicians who had worked together with a shared theoretical paradigm and health context. Further to this, the methodology using MRC guidance is constantly under review and has been criticized for its overemphasis on quantitative data collection (Grant et al., 2013).

Lovell et al. (2008) argue that intervention development using the MRC framework that draws on quantitative evidence does not necessarily provide good results. In fact, methodologies that place greater emphasis on the qualitative appraisal of results have been seen to improve outcomes following the intervention development phase (Richards et al., 2006). The sample size for the pilot was small, however the outcomes appeared to be good, both in terms of reducing symptoms of anxiety and depression at the end of treatment and in follow up. The aim was to develop an accessible intervention and to pilot this with a population to develop theoretical, practice and training requirements for an evidence informed intervention that may be more accessible to patients with complex depression. Further research should be conducted through a pragmatic randomized controlled trial recruiting from a wider number of people across multiple sites to ascertain the effectiveness of the intervention. Whilst the measures used appeared to demonstrate sustainable change, understanding the psychodynamic change process and impact of arts could be supported by using other measures relating to affect regulation (for example the Action and Acceptance Questionnaire II) and reflective functioning (for example the Reflective Functioning Questionnaire). Further to this, an in-depth qualitative appraisal and acceptability study should be conducted with the patient population to ascertain the relationship between referrer perceptions of acceptability and patient experiences of receiving ADIT.

Through the phased MRC intervention development model, the authors demonstrated a clear synergy between a talking based intervention and an arts-based intervention and successful integration of therapeutic structural elements (the time frame and focus of the intervention). The acceptability survey for mental health professionals showed that the majority of respondents perceived using image-making as part of the process within ADIT as increasing accessibility and outcomes. In summary, the MRC intervention development model used in this study helped clinician researchers to systematically reflect on and describe some complexities concerning theoretical cogency, acceptability and accessibility of an intervention for CD and the important role that art psychotherapy may play in providing accessible, effective treatment. The emergence of a range of arts-based evidence informed models of practice that are supported by empirical data (Uttley et al., 2015a,b) are promising and the authors hope this study will enable the introduction of more possibilities and choices for patients who may not access psychological treatment. Research for effectiveness of art psychotherapy for CD is at an early stage of development, however, using stringent methodologies to link practice with evidence to enhance practice and improve the mental health of vulnerable people has in this early phase of intervention development demonstrated good results.

## Data Availability Statement

The datasets presented in this study can be found in online repositories. The names of the repository/repositories and accession number(s) can be found in the article/supplementary material.

## Ethics Statement

The studies involving human participants were reviewed and approved by CNWL NHS Foundation Trust. The patients/participants provided their written informed consent to participate in this study.

## Author Contributions

DH-F coordinated the data collection, study design, and led the manuscript development process, carried-out the analysis of data and development and writing of the drafts and manuscripts and oversaw the development, the implementation of the study. DE led the intervention implementation, coordination, and contributed to the manuscript. DE and MO supported the intervention development and implementation and made the clinical scenario contributions. SS collated data and sociodemographic data and made contributions to the paper. All authors contributed to the article and approved the submitted version.

## Conflict of Interest

The authors declare that the research was conducted in the absence of any commercial or financial relationships that could be construed as a potential conflict of interest.
